# Comparative larvicidal efficacy and phytochemical profiling of selected Solanaceous hexane extracts against *Culex pipiens* and *Aedes aegypti*

**DOI:** 10.1038/s41598-026-68059-8

**Published:** 2026-09-05

**Authors:** Shaimaa H. Mohammed, Esraa A. Elhawary, Yasser A. El-Sayed, Mohamed M. Baz, Abdelfattah M. Selim, Mohammed H. Alruhaili, Hattan S. Gattan, Randa I. Eltaly

**Affiliations:** 1 Department of Zoology and Entomology, Faculty of Science, Al-Azhar University, Girls Branch, Cairo, Egypt; 2 Department of Pharmacognosy, Faculty of Pharmacy, Ain-Shams University, Cairo, 11566 Egypt; 3 Department of Entomology, Faculty of Science, Benha University, Benha, 13518 Egypt; 4 Department of Biology, Faculty of Education and Arts, Sohar University, Sohar, 311 Oman; 5 Department of Animal Medicine (Infectious Diseases), College of Veterinary Medicine, Benha University, Toukh, 13736 Egypt; 6 Department of Clinical Microbiology and Immunology, Faculty of Medicine, King Abdulaziz University, 21589 Jeddah, Saudi Arabia; 7 Department of Medical Laboratory Sciences, Faculty of Applied Medical Sciences, King Abdulaziz University, 22254 Jeddah, Saudi Arabia; 8 Special Infectious Agents Unit, King Fahad Medical Research Center, King Abdulaziz University, 21362 Jeddah, Saudi Arabia

**Keywords:** *Aedes aegypti*, *Culex pipiens*, Bio-insecticides, Larvicidal efficacy, UPLC/MS, Alkaloids, Withanolides, Solanaceae, Biochemistry, Chemical biology, Chemistry, Drug discovery, Plant sciences

## Abstract

Mosquito-borne diseases pose a major global health burden, and the effectiveness of conventional insecticides is increasingly limited by resistance and environmental concerns. This study provides a comparative assessment of the larvicidal activity and phytochemical composition of n-hexane extracts from four Solanaceous plants: *Capsicum annuum* (Cap.), *Datura stramonium* (Dat.), *Nicotiana tabacum* (To.), and *Withania somnifera* (Wt.). Volatile constituents were characterized using GC–MS analysis, and larvicidal activity was evaluated against *Culex pipiens* and *Aedes aegypti*, with LC₅₀ and LC₉₀ values determined at 24 and 48 h. post-treatment. All extracts showed concentration- and time-dependent toxicity, achieving near-complete mortality at 1000 ppm for *N. tabacum* and *D. stramonium*. *Nicotiana* tabacum showed the highest efficacy against *Cx. pipiens* (LC_50_ = 120.45 and 95.30 ppm, at 24 and 48 h), whereas *Ae. aegypti* showed comparatively lower susceptibility. Biochemical analyses revealed significant inhibition of acetylcholinesterase (AChE), *α*- and *β*-esterases, and GABA-transaminase, alongside an imbalance in antioxidant defenses, suggesting a multi-system physiological impact. GC–MS profiling identified distinct phytochemical compositions, particularly the alkaloid and fatty acid content identified in the more potent extracts. The four extracts contained a vast array of phytochemical components, reaching 110 components belonging to capsaicinoids and hydrocarbons for Cap; To. and Dat. were rich in fatty acids, particularly *n*-hexadecanoic and oleic acids; and Wt. exhibited a hydrocarbon-rich profile with cyclic and branched alkanes. Oleic acid was the only compound shared among three samples (Cap., To., and Dat.), whereas no compound was common across all samples. This study provides the first comparative assessment of four Solanaceous genera using integrated phytochemical, biochemical, and larvicidal analyses. The findings identify *N. tabacum* as the most promising candidate and establish a foundation for bioassay-guided development of plant-based bio-insecticides. This integrative approach offers a practical framework for screening botanical extracts for vector control programs, though further studies are needed to confirm the causal phytochemicals and mechanisms.

**Supplementary Information:**

The online version contains supplementary material available at https://doi.org/10.1038/s41598-026-68059-8.

## Introduction

Mosquito-borne diseases remain a major global public health concern, particularly in tropical and subtropical regions where environmental conditions favor mosquito survival and reproduction. *Culex pipiens* and *Aedes aegypti* are among the most important mosquito vectors responsible for transmitting several pathogens, including Rift Valley fever, West Nile virus, dengue, and chikungunya^1–6^.

The extensive and continuous use of synthetic insecticides has led to the development of resistance in mosquito populations, in addition to increasing concerns regarding environmental safety^7–9^. These limitations have encouraged the search for safer and more sustainable alternatives, with increasing attention directed toward plant-derived bioinsecticides. Current vector control strategies therefore aim to reduce mosquito populations by targeting different developmental stages, including larvae and adults, as well as limiting their capacity to transmit pathogens. Such integrated approaches are essential for disrupting disease transmission cycles and reducing infection rates^10^.

Medicinal plants represent a valuable source of bioactive compounds with both therapeutic and pesticidal properties, and are often considered to offer more environmentally compatible alternatives to conventional insecticides due to their biodegradability and potentially lower toxicity to non-target organisms, as suggested by several studies^11^. Within this botanical diversity, the family Solanaceae (the nightshades) emerges as a taxon of exceptional phytochemical significance. Comprising over 2,700 species, this family is renowned for its sophisticated chemical defense mechanisms, characterized by the synthesis of diverse bioactive secondary metabolites, including alkaloids, terpenoids, steroids, fatty acids, and capsaicinoids^12,13^. Despite their divergent morphological forms and economic uses, genera such as *Capsicum*, *Datura*, *Nicotiana*, and *Withania* share a common taxonomic lineage and a remarkable ability to produce potent toxins that serve as natural deterrents against herbivorous insects^14^.

Phytochemicals are generally classified into five major groups, including alkaloids, terpenoids, phenolics, steroidal lactones, fatty acids, growth regulators, and proteinase inhibitors. Within the Solanaceae family, these diverse phytochemical classes have been widely studied for their insecticidal potential, with alkaloids being among the most prominent. Early studies reported that plant-derived alkaloids such as nicotine, anabasine, and methyl anabasine were effective in inducing mortality in mosquito larvae. In parallel, other bioactive constituents such as capsaicinoids, withanolides, and various fatty acids have also been implicated in insecticidal activity. While plant-based compounds are often presented in the literature as environmentally safe due to their biodegradability, lower toxicity to non-target organisms, and reduced potential for resistance development in mosquito populations, it is important to note that these claims are based on literature-supported possibilities and require further validation under specific use conditions^15^.

A substantial body of research has investigated the insecticidal properties of the Solanaceae family, largely focusing on specific bioactive compounds such as nicotine and anabasine as potent neurotoxins capable of inducing rapid mortality in mosquito larvae^15^. Beyond acute toxicity, the biological activity of these plants extends to various sublethal impacts that disrupt growth, reproduction, and physiological homeostasis in several insect species^16^. However, despite this recognized potential and the increasing research on plant-derived agents, most studies have focused on individual species in isolation, often using different extraction solvents and bioassay conditions that complicate direct comparisons. Consequently, there remains a critical research gap in systematic comparative evaluations assessing the relative efficacy of different Solanaceae genera against major mosquito vectors under identical experimental conditions. This is particularly evident regarding the use of non-polar solvents like n-hexane, which effectively isolates the lipophilic bioactive constituents essential for pest control. The present study addresses this gap by providing a direct comparative assessment of four Solanaceous genera (*Capsicum*, *Datura*, *Nicotiana*, and *Withania*) using a common extraction solvent (*n*-hexane) and standardized bioassay protocols. This approach enables meaningful comparison of their relative potencies and facilitates the identification of the most promising candidates for further development. Importantly, the study integrates phytochemical profiling by GC–MS with biochemical and larvicidal assessments, offering a comprehensive framework for understanding the relationship between chemical composition and biological activity. Importantly, the present study investigates crude n-hexane extracts containing diverse phytochemical classes and does not aim to isolate or directly evaluate the mechanisms of individual alkaloids; rather, it seeks to correlate the overall phytochemical profiles with observed larvicidal activity^17^. Addressing this gap is vital for identifying the most promising botanical candidates and optimizing sustainable vector control strategies.

The biological activity of Solanaceae phytochemicals is attributed to their diverse chemical structures, which function as natural defense agents with broad-spectrum toxicity against a wide range of insect species. Their effects extend beyond acute lethality to include sublethal impacts across multiple levels of biological organization, highlighting their potential as effective agents for mosquito control^16,18,19^.

Among the prominent candidates in this family, *Capsicum annuum* (chili pepper) represents a widely distributed species of considerable agricultural and pharmacological importance. Beyond its global use as a food additive due to its characteristic pungency, *Capsicum* species are distinguished by the presence of capsaicinoids, a group of bioactive compounds responsible for their heat and synthesized in the fruit placenta through the condensation of vanillylamine with medium-chain fatty acids^17^. Major capsaicinoids, including capsaicin and its analogs, have been associated with diverse biological activities, ranging from antimicrobial and cytotoxic effects to analgesic and anti-inflammatory properties, in addition to their applications in medical treatments and pest control strategies^20^. In line with the growing interest in plant-derived bioactive compounds, these properties further support the potential of *Capsicum annuum* as a promising candidate in vector control research.

Within the Solanaceae family, *Datura stramonium* (thorn apple) has been widely recognized for its longstanding use in traditional medicine, where it has been employed in the management of pain, respiratory conditions, inflammatory disorders, and various skin ailments^21^. These applications are linked to its diverse array of bioactive constituents; however, its medicinal use is constrained by its well-documented toxicity, including neurotoxic and potential genotoxic effects. In parallel with its therapeutic relevance, *Datura* has also been investigated for its insecticidal properties. Evidence from previous studies indicates that its extracts exhibit activity against several insect pests, particularly mosquito vectors such as *Anopheles*, *Culex*, and *Aedes*, with notable larvicidal effects reported in multiple cases^22^. This combination of biological activity underscores the potential value of *Datura* in the development of plant-based strategies for mosquito control.

*Nicotiana tabacum*, a member of the Solanaceae family, is characterized by the presence of several secondary metabolites, including nicotine, terpenoids, flavonoids, and saponins. Among these constituents, nicotine is regarded as the principal compound responsible for its insecticidal activity, as it interferes with the insect nervous system, leading to respiratory impairment, paralysis, and eventual mortality^23^. Previous investigations have reported that tobacco-derived extracts can affect insect pests such as *Helicoverpa armigera*, although the observed mortality at certain concentrations, such as 43.3% at 10%, indicates only moderate effectiveness^24^. This level of activity suggests that the use of tobacco extract alone may not always provide sufficient control. Accordingly, recent studies have explored the use of combined plant extracts as a means to enhance insecticidal efficiency and optimize bioactivity while reducing the reliance on higher concentrations of a single plant source^25^.

*Withania somnifera*, commonly known as ashwagandha, is a medicinal plant belonging to the Solanaceae family. The roots of this species are rich in a variety of bioactive constituents, including alkaloids such as withanine, withasomnine, and somniferine, as well as steroidal lactones (withaferins and withanolides) and other phytosterols such as stigmasterol and sitoindosides. These compounds are associated with a wide range of pharmacological activities, including antimicrobial, anti-inflammatory, anti-tumor, anti-stress, anti-diabetic, cardioprotective, and neuroprotective effects^26^. Although the medicinal value of *W. somnifera* root extracts are well established, its potential as an insecticidal and insect growth regulatory agent has received comparatively limited attention, having been reported mainly against many insect species^27,28^. This underscores the potential value of further investigation into its role in insect control may be valuable within the context of plant-based bioinsecticide development.

This study provides the comparative assessment of four Solanaceous genera (*Capsicum*,* Datura*,* Nicotiana*, and *Withania*) using a unified extraction solvent (*n*-hexane) and standardized bioassay protocols. The principal contribution of this work lies in its integrative approach, which combines three complementary dimensions, including comprehensive phytochemical profiling by GC–MS to characterize the volatile and semi-volatile lipophilic constituents. comparative larvicidal bioassays against two major mosquito vectors (*Cx. pipiens* and *Ae. aegypti*) to evaluate biological efficacy; and furthermore, biochemical biomarker analyses (AChE, esterases, GABA-T, antioxidant enzymes, and oxidative stress markers) to elucidate potential mechanisms of action. This framework linking chemical composition to biological activity and physiological impact, provides a holistic understanding that has been lacking in previous studies, which typically addressed these dimensions in isolation. The integration of these complementary approaches establishes a practical foundation for bioassay-guided development of plant-based larvicides and supports the rational selection of the most promising candidates for further research and potential application in vector control programs.

In this context, the present study aims to comparatively evaluate the larvicidal activity of selected Solanaceae plant extracts, namely *Capsicum annuum* (chili pepper), *Datura stramonium* (thorn apple), *Nicotiana tabacum* (tobacco), and *Withania somnifera* (ashwagandha), against the larvae of *Culex pipiens* and *Aedes aegypti*. By employing n-hexane as a non-polar solvent, the study focuses on extracting lipophilic bioactive constituents and correlating their phytochemical profiles with the observed larvicidal efficacy. This approach is intended to identify the most effective plant-based candidates for mosquito control and to support the development of sustainable bioinsecticide strategies.

## Materials and methods

### Plant material collection and extract preparation

Mature leaves of *Capsicum annuum* (Cap.), *Datura stramonium* (Dat.), *Nicotiana tabacum* (To.), and *Withania somnifera* (Wt.) were collected from multiple sites within the agricultural fields of the Faculty of Agriculture, Qalyubiya Governorate, Egypt, during September–November 2025. Taxonomic identification of the plants was performed by Dr. Trease Labib, botanical consultant at the Egyptian Ministry of Agriculture. The samples were deposited in the herbarium of the Pharmacognosy Department, Faculty of Pharmacy, Ain Shams University, under accession codes *D. stramonium* (PHG-P-DS-588), *N. tabacum* (PHG-P-NT-589), *C. annuum* (PHG-P-CA-590), and *W. somnifera* (PHG-P-WS-591). Following collection, the plant material was thoroughly rinsed with running tap water to remove surface debris and then air-dried under shade at ambient temperature (27 ± 2 °C) until complete desiccation was achieved. The dehydrated leaves were subsequently ground into a fine powder using a stainless-steel electric grinder, and the powdered material was stored in airtight containers at room temperature to prevent moisture absorption until further use. For extraction, 40 g of the dried powdered plant material were extracted with 200 mL of HPLC-grade *n*-hexane (≥ 95%) using a Soxhlet apparatus for 5 to 7 h; the extraction temperature was maintained according to the boiling point of the solvent (approximately 69 °C) to ensure continuous cycling. The resulting crude extracts were subsequently concentrated under reduced pressure using a rotary evaporator at 40 °C and stored at 4 °C in amber glass vials for further bioassays^29^. The *n*-hexane extract was selected for GC–MS analysis because it is enriched in volatile and semi-volatile lipophilic constituents that are amenable to gas chromatographic separation. Highly polar, non-volatile constituents were not targeted by this analysis.

### Mosquito larvicidal assay

#### Rearing of *Culex pipiens* and *Aedes aegypti*

Laboratory colonies of *Culex pipiens* and *Aedes aegypti* mosquitoes were reared under standard laboratory conditions at 27 ± 2 °C, 75 ± 5% relative humidity, and a 12-hour light/12-hour dark period. Larvae were reared in enameled trays and fed a standard diet consisting of Tetramin^®^ fish food and finely ground bread at a 1:3 (w/w) ratio. Upon pupation, pupae were transferred to dechlorinated water cups and placed in mesh cages (35 × 35 × 40 cm) for adult emergence. Adult females were provided with blood meals from a hamster obtained from the animal facility of the Faculty of Science, Cairo University, a specialized unit for breeding and maintaining laboratory animals. All procedures involving animals were conducted in accordance with institutional and international guidelines for the care and use of laboratory animals and were approved by the Institutional Animal Care and Use Committee (IACUC), Cairo University (Approval ID: CUIF 49 − 20). For blood feeding, mosquitoes were allowed to feed directly on the hamster under controlled laboratory conditions. To minimize stress and discomfort, the hamster was anesthetized using isoflurane inhalation anesthesia following standard protocols. Both males and females were supplied continuously with a 10% sucrose solution as a carbohydrate source after a blood meal. To facilitate egg-laying, the cups were fitted with strips (or pieces) of moist sponge to serve as a suitable substrate for female *Ae. aegypti*, thus aiding egg-laying. In contrast, female house mosquitoes (*Cx. pipiens*) laid their egg clusters directly on the water surface. All developmental stages were consistently maintained under these controlled conditions to ensure a stable and uniform supply of experimental material^30^.

#### Larvicidal assay

The larvicidal activity of hexane extracts from *Capsicum annuum*,* Datura stramonium*,* Nicotiana tabacum*, and *Withania somnifera* was evaluated against the third-instar larvae of *Cx. pipiens* and *Ae. aegypti*. The extracts were first dissolved in a minimal volume of acetone as a carrier solvent. This solution was then serially diluted with dechlorinated distilled water to prepare the required concentrations (25, 50, 125, 250, 500, 1000 and 1500 ppm). The final acetone concentration in all test solutions, including the control, was maintained below 1% (v/v) to avoid any solvent-induced toxicity. For each bioassay, groups of twenty larvae were introduced into 250 mL glass beakers containing the respective concentrations. In parallel, a control group was maintained under identical conditions and treated with dechlorinated water. All experiments were performed in triplicate. Mortality was recorded after 24 and 48 h of exposure, in accordance with WHO guidelines^31^. Larval mortality was confirmed by the absence of movement upon gentle stimulation with a glass rod. Standard formulae were employed to calculate the percentage of mortality, and Abbott’s formula was used to determine corrected mortality values where necessary.

### Biochemical assessment and oxidative stress studies

For the biochemical assays, third-instar larvae were exposed to the calculated 24-h LC_50_ concentration of each extract for 24 h. Following exposure, surviving larvae were collected, pooled (approximately 0.5 g live weight, equivalent to 50–60 larvae), and homogenized for enzyme extraction. All biochemical analyses were performed on the surviving larvae only.

#### Enzyme extraction and preparation

Whole-body larvae were homogenized in liquid nitrogen and suspended in chilled sodium phosphate buffer (50 mM, pH 7). The homogenization was performed at 4 °C, followed by centrifugation at 10,000 × g for 10 min at 4 °C. The resulting supernatants were collected as the enzyme source for subsequent assays. Total protein content was quantified according to Bradford’s method^32^ sing bovine serum albumin (BSA) as a standard, with absorbance read at 595 nm.

#### Neuro-metabolic and digestive enzyme assays

Acetylcholinesterase (AChE) activity was determined using the modified Ellman’s method^33^ with acetylthiocholine iodide (ATChI) and DTNB at 412 nm. Non-specific esterases (α- and β-esterase) were assessed following Penilla et al.^34^ using naphthyl acetate substrates, with reactions terminated by o-dianisidine and measured at 570 nm. Gamma-aminobutyric acid transaminase (GABA-T) was quantified at 340 nm according to Boer and Bruinvels^35^. Regarding digestive enzymes, amylase activity was evaluated via the DNSA method^36^ at 540 nm, while lipase activity was measured by monitoring free fatty acid release from an olive oil emulsion at 550 nm^37^.

#### Oxidative stress and damage markers

Superoxide dismutase (SOD) and catalase (CAT) activities were determined following Nishikimi et al.^38^ and Aebi^39^, respectively. Glutathione S-transferase (GST) activity was measured by the conjugation of GSH with CDNB at 340 nm^40^, and reduced glutathione (GSH) levels were quantified as per^41^ measuring the absorbance at 412 nm. Oxidative damage was assessed through lipid peroxidation (LPO) by measuring malondialdehyde (MDA) levels as TBARS at 532 nm^42^. Additionally, total protein carbonyl (TPC) content was determined using the Levine et al.^43^ method to evaluate oxidative protein modification.

### Phytochemical identification

#### GC–MS analysis

For GC–MS analysis, the *n*-hexane extracts were filtered through a 0.45 μm syringe filter prior to injection. Each prepared sample was analyzed *via* a single GC–MS injection as one microliter of each extract was injected directly without further dilution. The *n*-hexane extracts of four Solanaceous plant (*Capsicum*,* Datura*,* Nicotiana*, and *Withania*) samples were injected into a gas chromatograph coupled to a mass spectrometer (Shimadzu GCMS-QP 2010, Kyoto, Japan) operating in EI mode at 70 eV, and mass spectrum acquisition was performed in the mass range of 35–500 amu at 70 eV and mass spectrum acquisition performed in the mass range of 35–500 amu. The instrument was equipped with an Rtx-5MS capillary column (30 m×0.25 mm i.d. ×0.25 μm film thickness: Restek, USA). One microliter sample was injected in a split injection mode with a split ratio of 10:1. Separation was achieved using an initial oven temperature at 45 °C for 2 min (isothermal), then gradually increased to 300 °C at a rate of 5 °C/min (ramp) and kept constant at 300 °C for another 5 min (isothermal). Helium was used as a carrier gas with a flow rate set at 1.4 mL/min. Injector temperature was maintained at 250 °C. Mass unit interface temperature was set at 280 °C and the ion source temperature was adjusted to 200 °C. Retention indices (RI) were calculated relative to a homologous series of *n*-alkanes (C8- C30) injected under the same GC conditions. Identification of the compounds was performed by comparing their mass spectra and retention indices with the data reported in NIST-17 and Wiley library databases^44^.

### Multivariate data analysis

The unsupervised principal component analysis (PCA) was performed using Unscrambler X 10.3 (CAMO SA, Oslo, Norway). A clustered heat map was built using NCSS. 12 software with Euclidean distance and the unweighted pair group method^45,46^. An illustrative Venn diagram was generated using the Biotools Venn diagram tool (available at: https://www.biotools.fr/misc/venny).

### Statistical analysis

Data were analyzed using SPSS (version 23, IBM, USA), where Probit analysis was applied to estimate lethal concentration (LC) values with 95% confidence intervals. In addition, a one-way ANOVA followed by Tukey’s HSD post hoc test was carried out to compare treatments and plant species, with statistical significance set at *P* < 0.05. Superscript letters in the tables indicate significant differences (*P* < 0.05) between treatments within the same column (a, b, c) and between concentrations within the same row (A, B, C). Principal component analysis (PCA) was performed using Unscrambler X 10.3 (CAMO SA, Oslo, Norway). A clustered heat map was created in NCSS 12 software, based on Euclidean distance and the unweighted pair group method.

## Results

### Evaluation of mosquito larvicidal effect

In the present study, the larvicidal efficacy of hexane extracts from four Solanaceous plants, *N. tabacum*,* D. stramonium*,* C. annuum*, and *W. somnifera*, was evaluated against *Cx. pipiens* and *Ae. aegypti* larvae across various concentrations. The results, recorded at 24 and 48 h post-treatment (PT), demonstrated that all tested extracts exhibited significant larvicidal activity, with clear concentration- and time-dependent toxicity (Tables 1, 2, 3 and 4). Statistical analysis (ANOVA followed by Tukey’s HSD post hoc test) confirmed significant differences between treatments (*P* < 0.05; exact p-values: *N. tabacum* vs. *W. somnifera*, *P* = 0.002; *N. tabacum* vs. *C. annuum*, *P* = 0.018; *D. stramonium* vs. *W. somnifera*, *P* = 0.031). All comparisons were statistically significant at *P* < 0.05. (as indicated by superscript letters in Tables 1, 2, 3 and 4). Results showed that *N. tabacum* and *D. stramonium* displayed the highest potency against *Cx. pipiens.* At 24 h PT, mortality rates for *N. tabacum*,* D. stramonium*,* C. annuum*, and *W. somnifera* at 500 ppm were 99%, 95%, 90%, and 87%, respectively. At the high concentration of 1000 ppm, complete mortality (100%) was achieved by the first three extracts, while *W. somnifera* reached 99%. By 48 h PT, all extracts achieved 100% mortality at 1000 ppm. At 500 ppm after 48 h, *N. tabacum*,* D. stramonium*, and *C. annuum* achieved 100% mortality, while *W. somnifera* reached 98% (Table 1), and significantly, all except *W. somnifera* reached total mortality even at the lower concentration of 500 ppm, highlighting the robust larvicidal potential of these Solanaceous species. At 48 h PT, all extracts achieved 100% mortality against *Cx. pipiens* at both 1000 and 1500 ppm, while against *Ae. aegypti*, all extracts achieved 100% mortality at 1500 ppm, while at 1000 ppm, mortality ranged from 95 to 100% depending on the extract (Table 2).

The results showed that extracts also displayed strong activity against *Ae. aegypti*, though slightly lower than that observed for *Cx. pipiens* (Table 2). At 24 h PT, mortality rates at 500 ppm were 83%, 72%, 92%, and 65% for *N. tabacum*,* D. stramonium*,* C. annuum*, and *W. somnifera*, respectively. At 1000 ppm, *N. tabacum* reached 100% mortality, followed by *D. stramonium* (96%) *C. annuum* (91%) and *W. somnifera* (86%). After 48 h of exposure, all extracts at 1500 ppm achieved 100% mortality, demonstrating the cumulative toxic effect over time. Compared to the control group (0% mortality), all tested extracts showed significant larvicidal activity.

**Fig. 1:**
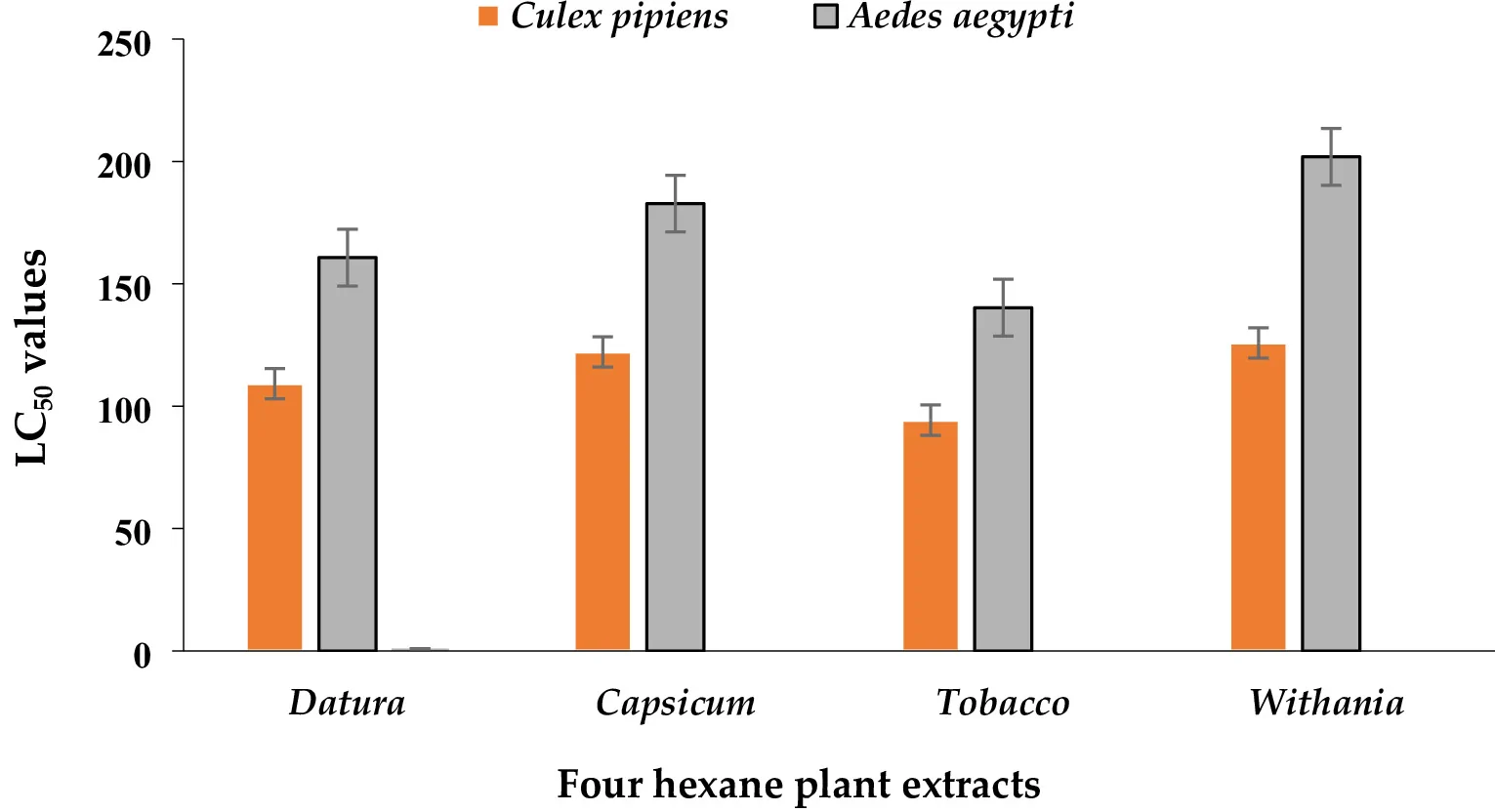
The mean number of larval mortalities induced by the effects of *Datura stramonium* extracts against 3rd larval instars of *Culex pipiens*, (**a**) and *Aedes aegypti* (**b**), 48 h post-exposure.

The calculated LC_50_ values further confirmed the superior toxicity of *N. tabacum* (Tables 3 and 4). Statistical comparison of LC_50_ values revealed that *N. tabacum* exhibited significantly lower LC_50_ values compared to all other extracts (*P* < 0.05, ANOVA followed by Tukey’s HSD post hoc test), confirming its significantly higher potency against both mosquito species (Tables 3 and 4). For *Cx. pipiens*, the LC_50_ values for *N. tabacum*,* D. stramonium*,* C. annuum*, and *W. somnifera* were 120.45, 139.36, 165.31, and 176.37 ppm at 24 h, decreasing to 95.30, 109.23, 122.16, and 125.85 ppm after 48 h, respectively. A similar trend was observed for *Ae. aegypti*, with *N. tabacum* recording the lowest LC_50_ values (176.97 and 141.26 ppm at 24 and 48 h, respectively). Comparatively, *Cx. pipiens* larvae exhibited greater susceptibility to the tested Solanaceous extracts than *Ae. aegypti* (Fig. 1).

### Assessment of biochemical parameters and oxidative stress

#### Neuro-metabolic and digestive enzyme inhibition

The biochemical analysis revealed that all Solanaceous hexane extracts induced a significant decline in enzymatic activities compared to the control group. Regarding neurotransmission and metabolic enzymes, the maximum inhibitory effect on AChE, α-EST, and β-EST was observed in larvae treated with *N. tabacum* (5.77, 1.41, and 1.89 µmol/min/mg protein, respectively), followed by *D. stramonium* (7.33, 1.79, and 2.40 µmol/min/mg protein, respectively) (Table 5). Furthermore, *N. tabacum* significantly restricted the activities of GABA-T, amylase, and lipase, recording 2.73, 7.74, and 1.15 units, respectively, which was markedly lower than the levels observed with other plant extracts (Table 6). These results suggest that the tested extracts, particularly tobacco, severely disrupt the nervous and digestive functions of the larvae.

#### Impact on detoxification and oxidative stress markers

The study further indicated that all plant extracts were highly effective at inhibiting detoxification enzymes and modulating oxidative stress markers in the treated mosquitoes. *N. tabacum* extract exhibited the most pronounced inhibitory effect, significantly reducing the levels of SOD, CAT, GST, and GSH, while altering damage markers such as LPO and TPC. The recorded values for these parameters in the tobacco-treated group were 1.50, 25.91, 2.25, 0.97, 8.56, and 13.34 units, respectively (Table 7). This suppression of the antioxidant defense system, coupled with the inhibition of detoxification enzymes, strongly suggests a multi-target toxicity of the Solanaceous phytochemicals against the larvae. However, further mechanistic studies, including enzyme kinetics and gene expression analyses, are required to confirm the precise pathways involved and to identify the primary molecular targets.

### Phytochemical characterization of the metabolites through GC–MS analysis

The phytochemical profiling of the *n*-hexane extracts of *Capsicum annuum* (Cap.), *Datura stramonium* (Dat.), *Nicotiana tabacum* (To.), and *Withania somnifera* (Wt.) resulted in the identification and quantification of one hundred and ten components as listed in Table 8. The GC/MS chromatograms for the four *n*-hexane samples were illustrated in Suppl. Figure 1–4. The most abundant phytochemical classes were varied between samples where *C. annuum* was dominated by capsaicinoids and hydrocarbons. In addition, *N. tabacum* and Dat. shared a common phytochemical profile dominated by fatty acids, particularly *n*-hexadecanoic and oleic acids. In contrast, the *W. somnifera* sample exhibited a hydrocarbon-rich profile with cyclic and branched alkanes. Oleic acid was the only compound shared among three samples (Cap, To, and Dat), whereas no compound was common across all samples.

For *capsicum n*-hexane extract, fifty compounds were detected with a % identification of 92.56%, where the abundant components were capsaicin (15.46%), dihydrocapsaicin (14.31%), dodecane (8.27%), 3-methylnonane (4.63%), and tritetracontane (4.18%) as detailed in Table 8. Moreover, the tobacco (*N. tabacum*) *n*-hexane extract presented twelve components with a % identification of 99.20%, and it was predominated by (S)−3-(1-methyl-2-pyrrolidinyl) pyridine as a characteristic major peak (85.96%), followed by *n*-hexadecanoic acid (6.77%) with other minor components. Likewise, the *Datura* (Dat.) *n*-hexane extract showed twelve components (98.36%), where *n*-hexadecanoic acid (53.81%) appeared as a dominant peak in addition to oleic acid (20.29%), octadecanoic acid (stearic acid) (4.25%), hexadecanoic acid, methyl ester (4.11%), (S)−3-(1-methyl-2-pyrrolidinyl)-pyridine (4.00%), and (E)−9-octadecenoic acid, methyl ester (3.26%). Lastly, *Withania* (Wt.) *n*-hexane extract was presented by forty-nine components (74.54%) with its main components as octane (16.91%), 1,3-dimethyl-adamantane (15.04), cis-1,3-dimethyl-cyclohexane (6.55%), 2-methyl-heptane (5.65%), and decahydro-2,6-dimethyl-naphthalene (5.01%).

**Fig. 2:**
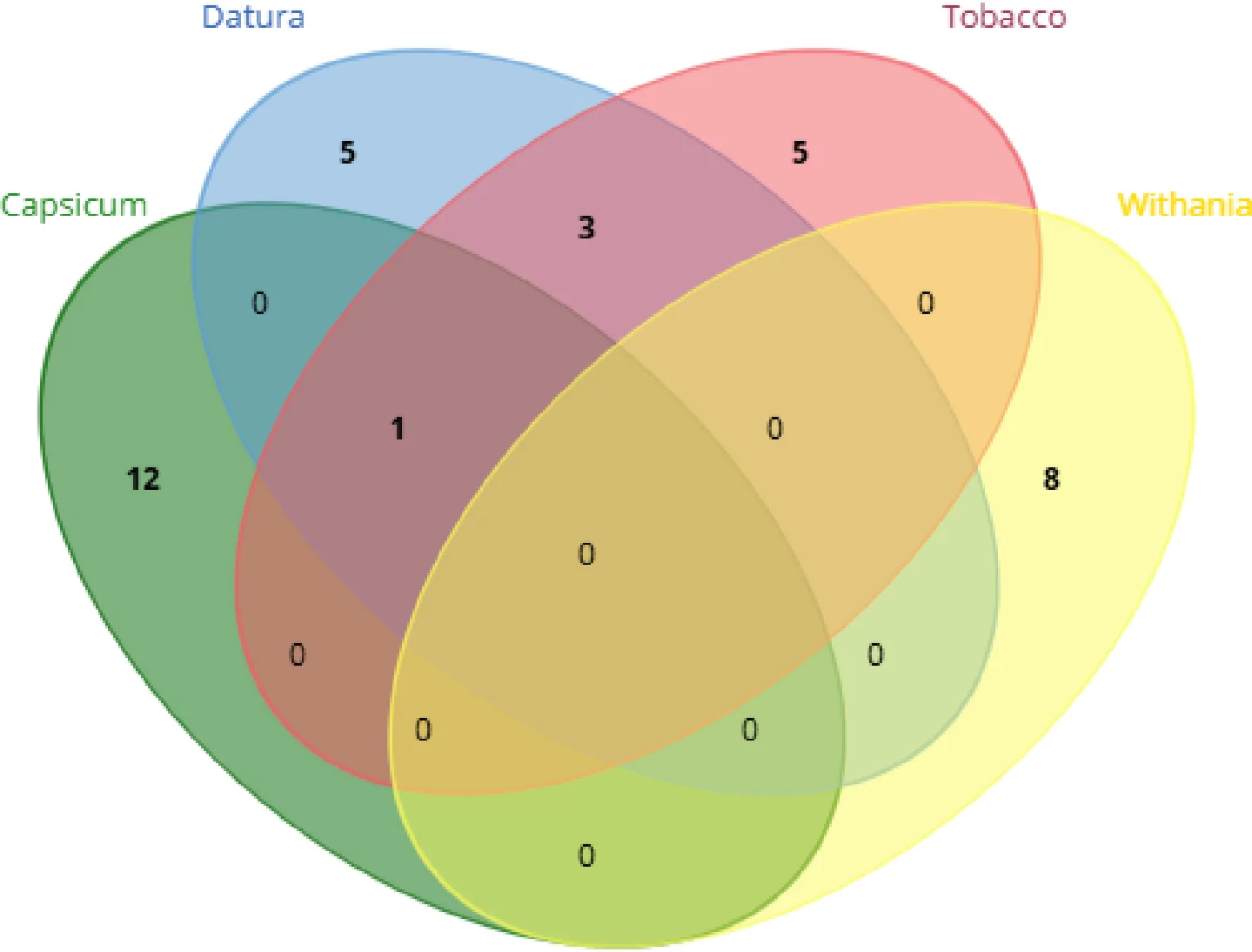
Venn diagram illustrates the distribution of identified metabolites among *Capsicum*, *Datura*, *Nicotiana*, *Withania n*-hexane extracts through GC–MS analysis. Numbers indicate the count of unique and shared metabolites.

Although the selected four plants belonged to the same family (Solanaceae), they carried distinct phytochemical profiles as illustrated in the Venn diagram (Fig. 2), interlinking their identified components. The overlapping metabolite sections were derived from the metabolites identified from their GCMS runs. The central part of the Venn diagram showed zero metabolites, indicating no common component between the samples. In addition, each sample carried a certain number of characteristic metabolites listed as 12 Cap., 5 Dat., 5 To., and 8 Wt. Certain components like oleic acid appeared as a common metabolite for Cap., To., and Dat., while (S)−3-(1-methyl-2-pyrrolidinyl)-pyridine, *n*-hexadecanoic acid, hexadecanoic acid methyl ester, and octadecanoic acid were shared between To. and Dat. Moreover, Cap. and Wt. shared no distinct component while they overlapped in containing a number of hydrocarbons.

### Multivariate data analysis

**Fig. 3:**
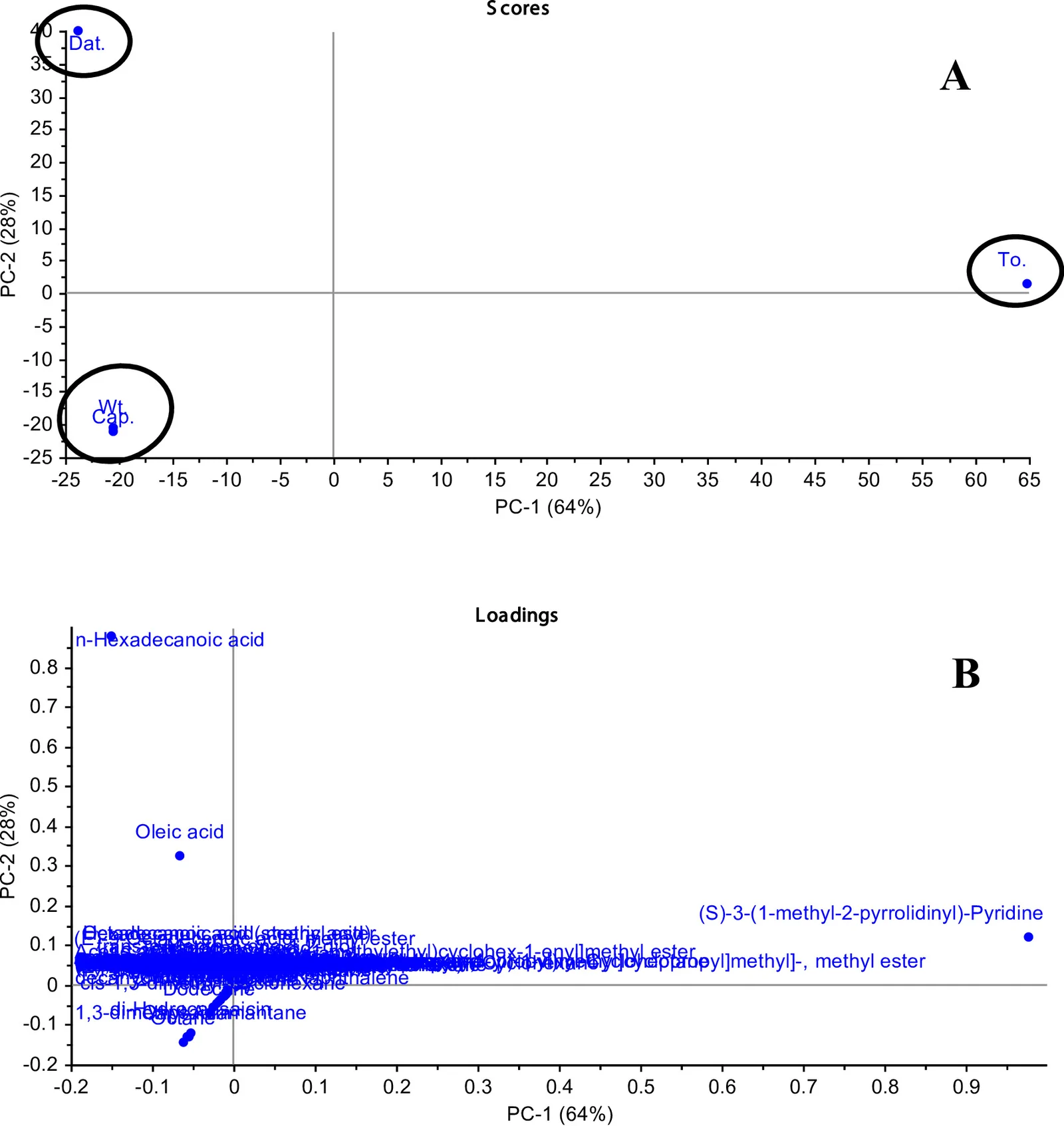
(**A**) Score plot of PC_1_ *versus* PC_2_ of the GC–MS identified metabolites from *Capsicum*, *Datura*, *Nicotiana*, *Withania n*-hexane extracts (area % as a variable). (**B**) Loading plot for PC1 and PC2 contributing metabolites and their assignments (area % as variable). Dat.: *Datura stramonium*,* Cap.: Capsicum annuum*, To.: *Nicotiana tabacum*,* Wt.: Withania somnifera*.

**Fig. 4:**
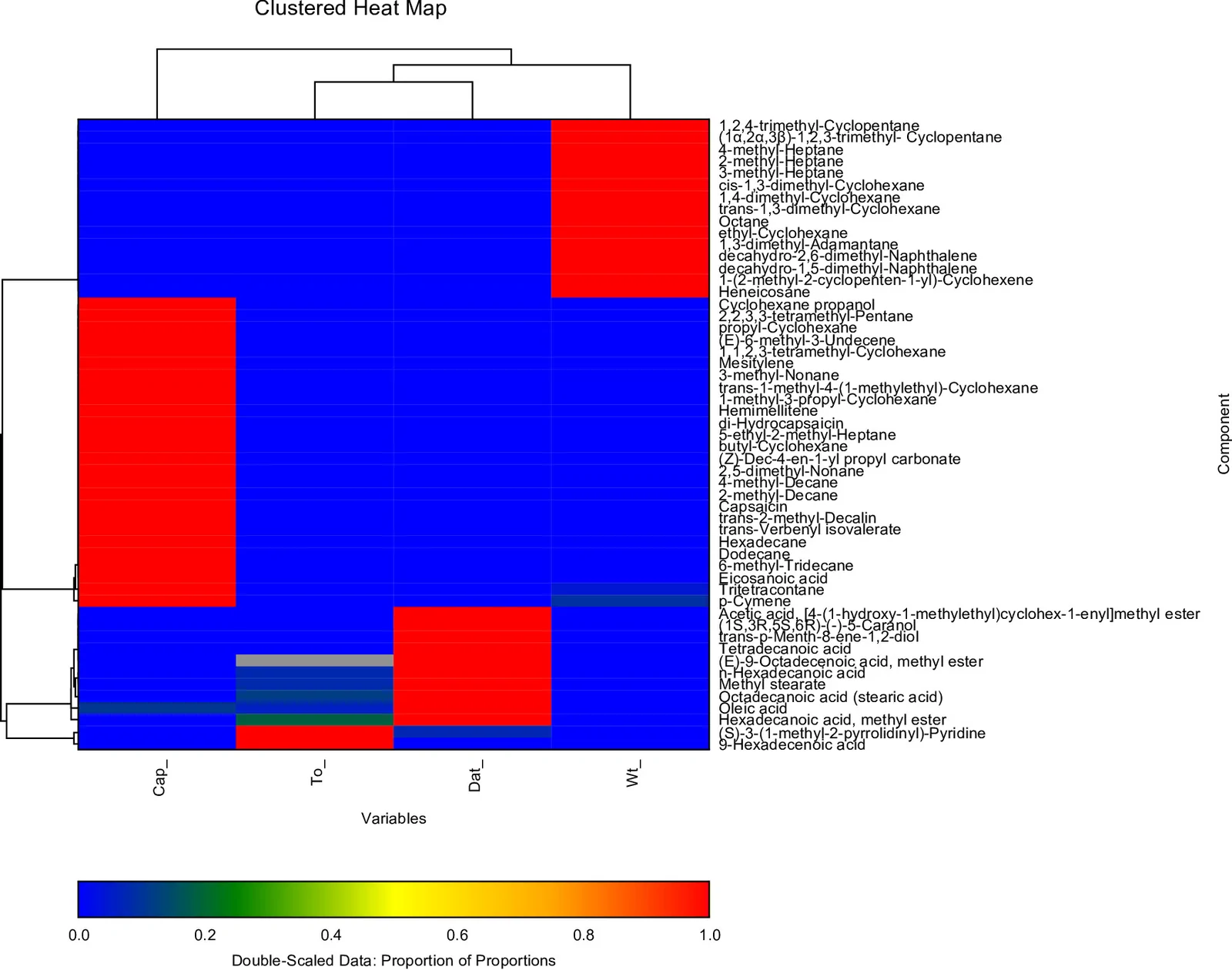
Clustered heat map showing the GC–MS identified components from *Capsicum*, *Datura*, *Nicotiana*, *Withania n*-hexane extracts. A heat map was constructed using Euclidean distance and the unweighted group method (components with % composition ≥ 1% were included). Dat.: *Datura stramonium*, Cap.: *Capsicum annuum*, To.: *Nicotiana tabacum*,* Wt.: Withania somnifera*.

The clustering pattern for the analyzed samples was detected through principal component analysis (PCA) as an unsupervised technique. The PCA score plot (Fig. 3A), used as an exploratory visualization tool, showed the relative positioning of the four samples based on their phytochemical composition. *D. stramonium* (Dat.) was located in the upper left quadrant, while *N. tabacum* (To.) was positioned at the intersection between the upper and lower right quadrants. *C. annuum* (Cap.) and *W. somnifera *(Wt.) showed overlap in the left lower quadrant, reflecting their similar hydrocarbon-rich profiles. It should be noted that with only four samples, PCA serves as a qualitative visualization rather than a statistically validated clustering analysis. This differential positioning pattern could be explained by the loading plot results shown in Fig. 3B, where the presence of unique phytochemical components for Dat. and To. resulted in their distinct separation from the others, while the overlapping hydrocarbon-rich profiles of Wt. and Cap. resulted in their proximity on the score plot. The clustered heat map (Fig. 4), including components with % composition ≥ 1, is presented as a visual representation of compositional differences among the four extracts. The color gradient (blue for lowest % composition to red for highest % composition) illustrates the relative abundance of each metabolite. The dendrogram shows the hierarchical clustering based on Euclidean distance and serves as a visualization tool to support the GC–MS findings, rather than a statistically validated clustering analysis. The overlap between Wt. and Cap. on the PCA score plot is attributed to both extracts being predominantly rich in hydrocarbon-class compounds (including various alkanes, cycloalkanes, and branched hydrocarbons), even though they do not share identical individual compounds. This compositional similarity at the class level explains their proximal positioning, despite the absence of shared specific metabolites.

## Discussion

The extensive use of synthetic insecticides in mosquito control has resulted in several limitations, most notably the development of resistance in mosquito populations^47^. This challenge has prompted increasing interest in alternative control strategies that combine effectiveness with environmental safety. In this context, biopesticides have emerged as promising candidates due to their lower toxicity and reduced ecological impact. Accumulating evidence indicates that many plant species possess larvicidal properties, supporting their potential as sustainable alternatives to conventional insecticides^7^.

Plant-derived extracts are widely utilized in agriculture and public health for the management of pests affecting humans and animals^48^. Their efficacy is largely attributed to the presence of diverse bioactive secondary metabolites with multiple biological activities. In addition to their effectiveness, these natural products tend to degrade into less harmful compounds, which enhances their suitability for integration into mosquito management programs^49^. Accordingly, plant-based insecticides have demonstrated considerable potential in reducing mosquito populations compared with synthetic counterparts^50^.

The present study evaluated the larvicidal potential of hexane extracts obtained from four Solanaceae plants (*Capsicum annuum*, *Datura stramonium*, *Nicotiana tabacum*, and *Withania somnifera*) against two medically important mosquito vectors, *Culex pipiens* and *Aedes aegypti*. Mosquitoes are widely recognized as major vectors of numerous diseases with global distribution, which makes their control particularly challenging^51^. Consequently, increasing attention has been directed toward the exploration of plant-based insecticides derived from locally and widely available plant species as alternative control options^10^.

The present study demonstrated that hexane extracts of four Solanaceous plants (*Capsicum annuum*,* Datura stramonium*,* Nicotiana tabacum*, and *Withania somnifera*) exhibited significant larvicidal activity against *Cx. pipiens* and *Ae. aegypti* in a clear concentration- and time-dependent manner. Based on the calculated LC₅₀ values (the most reliable measure of potency), *N. tabacum* demonstrated the highest toxicity (LC₅₀ = 120.45 and 95.30 ppm for *Cx. pipiens*, and 176.97 and 141.26 ppm for *Ae. aegypti* at 24 and 48 h., respectively), followed by *D. stramonium*,* C. annuum*, and *W. somnifera.* At a concentration of 500 ppm against *Ae. aegypti*, *C. annuum* showed higher mortality (92%) than *D. stramonium* (72%); however, the overall LC₅₀-based ranking provides a more comprehensive measure of potency across all concentrations. High mortality rates were observed at 500–1000 ppm, with mortality exceeding 95% for most extracts after 48 h. LC₅₀ values further confirmed the superior efficacy of *N. tabacum* (120.45 and 95.30 ppm for *Cx. pipiens*, and 176.97 and 141.26 ppm for *Ae. aegypti* at 24 and 48 h, respectively), with *Cx. pipiens* showing greater susceptibility than *Ae. aegypti*. The data revealed that the common house mosquito (*Culex pipiens*) was more sensitive to the tested extracts than the *Aedes aegypti* mosquito. This contrasting sensitivity is a highly significant observation. It may be attributed to species-specific differences in detoxification enzyme systems (such as glutathione transporters and esterases) or to physiological barriers between the two species, as suggested by previous studies^52^. However, this hypothesis requires further investigation, as the present study did not directly compare baseline enzyme levels or detoxification capacity between the two species.

The observed larvicidal activity increased significantly with both concentration and exposure time, indicating a clear concentration–response relationship. This pattern can be explained by the presence of bioactive secondary metabolites, whose composition and extraction efficiency play a critical role in determining insecticidal activity. In addition, increasing concentration may enhance the availability of active compounds, thereby amplifying their biological effect. Similar trends have been reported in previous studies, which consistently demonstrated a positive relationship between larvicidal efficacy and concentration^52,53^.

The effectiveness of the extracts can also be linked to the use of a non-polar solvent. The present findings indicate that *n*-hexane is particularly efficient in extracting bioactive phytochemicals associated with larvicidal activity, likely due to the higher solubility of non-polar secondary metabolites in such solvents. This results in extracts with greater biological potency. Previous studies have similarly shown that the efficacy of plant extracts increases as solvent polarity decreases, with *n*-hexane fractions often exhibiting stronger insecticidal activity than aqueous or methanolic extracts. This trend, observed across multiple plant species, may indicate that key mosquitocidal compounds are predominantly non-polar. Therefore, the high larvicidal activity recorded in this study further supports the effectiveness of *n*-hexane extracts as a promising source of botanical insecticides^54–56^.

In line with the present study, previous findings have highlighted the pronounced insecticidal efficacy of *N. tabacum*, which is largely attributed to its nicotine content acting as a synaptic toxin that disrupts neural transmission and induces paralysis. This agrees with the current results, where *N. tabacum* exhibited the highest larvicidal activity and the strongest inhibition of acetylcholinesterase (AChE), confirming its dominant neurotoxic effect. In contrast, *D. stramonium* has been reported to show comparatively lower and delayed toxicity, which is consistent with the present findings where its larvicidal and biochemical effects were less pronounced than those of tobacco extract^15^.

The superior larvicidal activity of *N. tabacum* is likely attributable to its high alkaloid content, particularly nicotine (85.96% by GC–MS), which acts as a potent neurotoxin by competitively inhibiting acetylcholinesterase and disrupting cholinergic neurotransmission. This neurotoxic effect is further amplified by the synergistic action of fatty acids (e.g., *n*-hexadecanoic acid, 6.77%) and other minor constituents, which may facilitate cuticular penetration and enhance the overall bioavailability of the active compounds.

In agreement with the present study, earlier investigations have demonstrated that *N. tabacum* extracts exhibit a clear concentration- and time-dependent increase in larval mortality. Reported mortality rates ranged from 3.33 to 23.33% and 10–56.67% at 24 and 48 h, respectively, for lower concentrations (4–15%), while higher concentrations produced substantially greater effects, reaching up to 80% at 72 h and 93.33% at 96 h. This pattern reflects a cumulative toxic effect over time and is consistent with the current findings, where larval mortality increased markedly with both concentration and exposure duration^25^. Such observations agree with previous reports indicating that mortality rises proportionally with extract concentration due to the increased availability of active compounds^57^. The strong bioactivity of *N. tabacum* is largely attributed to its rich composition of secondary metabolites, including nicotine, saponins, and flavonoids^16^. These compounds have been shown to interfere with feeding behavior, disrupt digestion, and impair nutrient absorption, ultimately leading to larval death^58^.

A partial similarity with the present study was observed, where root extracts of *Withania somnifera* exhibited pronounced insecticidal and growth-disrupting effects on *Sarcophaga ruficornis*. These effects included dose-dependent larval and pupal mortality, delayed pupariation, prolonged developmental duration, and reduced adult emergence, particularly in younger instars^28^. Comparable outcomes have been reported in other dipteran species following exposure to plant extracts and insect growth regulators^59^. Such effects are likely attributed to bioactive compounds, including withanolides, which mimic juvenile hormone activity and disrupt the hormonal regulation of moulting and metamorphosis^26,60–63^.

Also, a partial similarity with the present study was observed, where *C. annuum* extracts exhibited strong insecticidal activity against mosquito and housefly larvae, achieving high mortality rates within 24 h of exposure. The larvicidal efficacy increased with concentration, with petroleum ether extracts showing the highest potency, consistent with previous findings on *Culex quinquefasciatus* and *Anopheles stephensi*^64,65^. These effects are attributed to bioactive secondary metabolites that interfere with physiological processes, including significant inhibition of key digestive enzymes such as protease, amylase, and lipase. Such biochemical disruption leads to metabolic imbalance, impaired growth, and eventual larval mortality, supporting the potential of plant-derived compounds as effective bioinsecticides^66^.

In addition to larval mortality, the present findings indicate that exposure to Solanaceae extracts induces profound biochemical and physiological disturbances in mosquito larvae. The consistent inhibition of key enzymes relative to the control suggests that the extracts interfere with essential metabolic and detoxification pathways rather than acting through a single isolated target. This multi-level disruption is particularly important in explaining the overall toxicity observed in treated larvae.

Among the tested plants, *N. tabacum* exhibited the most pronounced inhibitory effects on acetylcholinesterase (AChE), *α*-esterase, and *β*-esterase activities. The suppression of AChE activity is especially significant, as it directly affects synaptic transmission through accumulation of acetylcholine, ultimately leading to neurophysiological dysfunction^67^. This neurotoxic action is likely a major contributor to the rapid onset of paralysis and mortality observed in larvae exposed to tobacco extract. Additionally, the inhibition of *α*- and *β*-esterases is suggestive of disruption of detoxification pathways, which may increase the susceptibility of larvae to the toxic effects of the phytochemicals. The significant reduction in GABA-T activity indicates interference with GABAergic neurotransmission, which plays a crucial role in regulating neuronal excitability in insects^35^. Furthermore, the observed imbalance in antioxidant defences (reduced SOD, CAT, GST, and GSH) coupled with elevated oxidative damage markers (LPO and TPC) suggests that oxidative stress is a key component of the toxic mechanism. This oxidative damage likely compromises cellular integrity through membrane lipid degradation, which can accelerate physiological collapse in treated larvae. The simultaneous disruption of multiple physiological pathways indicates that the plant extracts do not act on a single biochemical target but rather induce systemic toxicity through a complex interplay of mechanisms. However, further studies, including enzyme kinetics, gene expression analyses, and molecular docking, are needed to confirm the precise molecular targets and pathways involved. The comparatively weaker inhibition recorded for *D. stramonium* and *C. annuum* appears to reflect differences in the concentration or potency of neuroactive phytochemicals among the tested species, which is consistent with the variation observed in larvicidal activity.

The phytochemical profiling of *N. tabacum* and *D. stramonium* showed a predominance of fatty acids and alkaloids, which is consistent with the observed neurotoxic effects, as these compound classes are known to interfere with nervous system function in insects^25^. Specifically, the potent inhibition of AChE by the tobacco extract may be attributed to its high alkaloid content (particularly nicotine, identified at 85.96% by GC–MS), as alkaloids are known to act as competitive inhibitors at the synaptic level^25^. However, it is important to note that the present study investigated crude *n*-hexane extracts and did not perform bioassay-guided fractionation; therefore, determining which specific components and their individual concentrations are responsible for the observed larvicidal activity requires further investigation. The current findings establish a correlation between the overall phytochemical profiles and bioactivity, providing a foundation for future studies aimed at isolating and characterizing the active principles and confirming their causal role in the observed toxicity^25^. *Nicotiana tabacum* and *D. stramonium* phytochemicals revealed a predominance of biologically active compounds like alkaloids and fatty acids, which correlates well with the observed biochemical disruptions. Specifically, the high nicotine content (85.96%) in *N. tabacum* is consistent with its potent AChE inhibitory activity (5.77 µmol/min/mg protein), as nicotine is known to act as a competitive inhibitor at the cholinergic synapse^8^. Similarly, the abundance of *n*-hexadecanoic acid (53.81%) and oleic acid (20.29%) in *D. stramonium* may account for its notable inhibition of *α*- and *β*-esterases (1.79 and 2.40 µmol/min/mg protein, respectively), as these fatty acids are known to disrupt membrane integrity and interfere with lipid metabolism^13^. Furthermore, the presence of diverse hydrocarbons in W. somnifera may contribute to its modulatory effects on antioxidant enzymes and oxidative stress markers, as hydrocarbon metabolism is known to generate reactive oxygen species and trigger antioxidant responses^28^.

Beyond neurotoxicity, the reduction in enzymes involved in digestion and metabolism, including GABA-T, amylase, and lipase, points to a broader physiological impairment. The simultaneous disruption of energy metabolism and neural regulation indicates that the plant extracts do not act on a single biochemical target but rather induce systemic toxicity. In this context, the stronger effects of *N. tabacum* suggest a more complex or synergistic phytochemical profile capable of affecting multiple physiological pathways.

A further important observation is the marked imbalance in antioxidant defense systems. The significant reduction in SOD, CAT, GST, and GSH activities, accompanied by elevated lipid peroxidation, indicates that oxidative stress is a key component of the toxic mechanism. This oxidative damage likely compromises cellular integrity through membrane lipid degradation, which can accelerate physiological collapse in treated larvae. Importantly, this redox imbalance does not appear as a secondary consequence alone but is likely integrated with neurotoxic and metabolic effects, reinforcing the multi-target nature of plant extract toxicity^68^.

Overall, these biochemical disruptions suggest that the larvicidal activity of Solanaceae extracts is mediated through combined mechanisms involving neurotoxicity, metabolic inhibition, and oxidative stress. The dominance of these effects in *N. tabacum* further supports the hypothesis that its superior larvicidal efficacy is associated with a richer or more potent profile of bioactive secondary metabolites capable of acting on multiple biological systems simultaneously.

The insecticidal effects of plant-based insecticides are linked to several interacting mechanisms that reflect their diverse chemical constituents. The phytochemical classes identified in our study, including alkaloids, fatty acids, capsaicinoids, and hydrocarbons, have been previously associated with insecticidal activity through various mechanisms. Alkaloids such as nicotine are known neurotoxins that interfere with synaptic transmission, while fatty acids can disrupt membrane integrity and cuticular penetration^69^. Capsaicinoids have been reported to cause metabolic disruption, and hydrocarbons may contribute to the lipophilic properties that facilitate penetration through the insect cuticle^20^.

Moreover, the lipophilic character of these compounds facilitates their penetration through insect cuticle and tissues, where they compromise membrane integrity and cause leakage of cellular contents^69^. Another important pathway of toxicity involves oxidative stress, which results from excessive generation of reactive oxygen species that damage essential biomolecules such as lipids, proteins, and nucleic acids^70^.

In addition, exposure to sublethal doses of these compounds can trigger notable physiological and biochemical alterations, particularly in key enzyme systems^71^. Changes in detoxification, neural function, and oxidative stress-related enzymes in mosquito larvae indicate a coordinated response to chemical stress. For instance, reduced activity of glutathione S-transferase (GST) is indicative of a weakened detoxification capacity and increased sensitivity to toxic substances, whereas increased GST activity may reflect an adaptive response aimed at enhancing detoxification^72^. Likewise, fluctuations in acetylcholinesterase activity demonstrate disturbance of cholinergic signaling; inhibition is associated with neurotoxic effects, while elevated levels may indicate compensatory regulation by the organism^73,74^.

Additionally, variations in detoxification and digestive enzymes, such as esterases and phosphatases, are consistent with a biochemical impact of the plant extract. Altered α-esterase activity in certain treatments could reflect the activation of alternative detoxification pathways, while relatively stable β-esterase activity indicates a more selective enzymatic response to exposure^75,76^. Overall, these findings highlight that the insecticidal effects of essential oils are mediated through a complex interplay of neurotoxicity, metabolic disruption, and oxidative stress.

The comparative phytochemical profiling conducted in this study provides a chemical basis for the observed variations in larvicidal efficacy among the four Solanaceous plants. Although sharing a common taxonomic origin, the distinct phytochemical landscapes of *Capsicum annuum* (Cap.), *Datura stramonium* (Dat.), *Nicotiana tabacum* (To.), and *Withania somnifera* (Wt.) explain their differential biological impacts. Specifically, the similar profile of To. and Dat., characterized by a high abundance of fatty acids, notably n-hexadecanoic and oleic acids, correlates with their superior toxic effects, as these lipophilic compounds are known to facilitate cuticular penetration and disrupt membrane integrity in mosquito larvae.

In contrast, the hydrocarbon-rich profile of Wt. and the capsaicinoid-dominated composition of Cap. resulted in different modes of action or lower potency. Our findings are consistent with recent literature; for instance, the dominance of capsaicinoids and sterols in *C. annuum* has been previously linked to its specific insecticidal properties^77^. Similarly, the effectiveness of the non-polar *n*-hexane extracts used in this study is supported by evidence that such solvents are most efficient at extracting fatty acids, sterols, and triterpenoids from *D. stramonium*, whereas polar alkaloids typically require different extraction methods. In another study, the *n*-hexane extract of three Ethiopian *C. annuum* varieties were analyzed and eighty components were traced. The major components included capsaicin, dihydrocapsaicin, vitamin E and (24R)-stigmast-5-en-3*β*-ol^78^. Da*tura stramonium* is phytochemically characterized by tropane alkaloids and diverse fatty acid profiles. An investigation of different *D. stramonium* seed extracts, using solvents of varying polarities, showed that the non-polar extracts (*n*-hexane, ether, chloroform) are rich in fatty acids, sterols, glycosides, triterpenoids, and phenolic compounds, while polar extracts (methanol, ethanol, water) are predominantly abundant in alkaloids^79^.

Likewise, the GC–MS profile of *Nicotiana tabacum* is dominated by alkaloids, particularly nicotine, which may be subjected to extensive transformation during post-harvest processing. A comparative study examined the PT76 variety consumed as khaini in India demonstrated that green leaves contain twenty-five compounds including nicotine, naphthalene, neophytadiene, phytol, stigmasterol and *β*-sitosterol. In contrast, cured leaves exhibited forty-two compounds with nicotine as the dominant constituent, in addition to potentially harmful compounds such as 1,2,3,6-tetrahydro-2,3′-bipyridine (1.19%), 4-nitroso-3-(pyrrolidin-1-yl)-phenol and 1-methyl-5-(pyridin-3-yl)-pyrrolidin-2-one^80^. The essential oil of *N. tabacum* leaves from Lebanon were analyzed where nineteen volatile constituents were detected with pentadecanal, biosol, solanone, thymol, damascenone and *β*-caryophyllene were the major components^81^. In addition, *Withania somnifera* (ashwagandha) phytochemical components were screened employing GC–MS analysis of its leaf essential oil where the key compounds include 2-methoxy-4-vinylphenol, hexadecanoic acid, N,N-*bis*-(2-hydroxyethyl) dodecanamide, 1,2-benzene dicarboxylic acid diethyl ester and oxacycloheptadec-8-en-2-one^82^. Ultimately, the absence of a single common chemical component across all samples, except for oleic acid in three, suggests that the larvicidal potential of these extracts is likely driven by the interplay of diverse secondary metabolites rather than a single molecule. This could involve additive or synergistic effects among the various phytochemical classes^72^, though this hypothesis requires further investigation through bioassay-guided fractionation and combination studies.

It is important to note that although GC–MS analysis identified several compounds potentially associated with the observed biological activity, this study did not perform bioassay-guided fractionation or histopathological examinations. Therefore, identifying the specific components directly responsible for the larvicidal and biochemical effects requires further investigation. Nevertheless, the observed correlations, particularly the association between high alkaloid (nicotine) content and strong acetylcholinesterase inhibition, the relationship between fatty acid abundance and esterase inhibition, and the link between hydrocarbon-rich compositions and oxidative stress, provide a solid foundation for future hypothesis-driven mechanistic studies.

It is important to note that the present study was conducted under controlled laboratory conditions, which do not fully replicate the complex environmental factors encountered in natural mosquito breeding sites. Factors such as UV radiation, water chemistry (pH, hardness, organic matter), temperature fluctuations, rainfall, and formulation stability may significantly influence the field performance of these botanical extracts. Therefore, while laboratory results are promising, field evaluations are essential to assess the practical efficacy of these extracts under natural conditions and to determine optimal application strategies.

These limitations open several avenues for future research. Bioassay-guided fractionation should be employed to isolate and characterize the active components, determine their individual LC_50_ values, and investigate their potential synergistic or antagonistic interactions. Additionally, in-silico molecular docking studies are recommended to investigate the potential binding interactions between the identified phytochemicals (particularly alkaloids such as nicotine, capsaicinoids, and fatty acids) and key target enzymes including acetylcholinesterase (AChE), esterases, and GABA-transaminase. Such computational studies would complement the biochemical findings and provide valuable insights for identifying potential lead compounds. Furthermore, field evaluations are necessary to assess the efficacy of these extracts under natural environmental conditions, alongside comprehensive ecotoxicological assessments to evaluate effects on non-target organisms. Finally, formulation development and resistance management studies should be conducted to explore the potential for creating stable bio-insecticide products suitable for integrated vector management programs.

Future studies should aim to: (i) confirm the causal role of specific compounds through bioassay-guided fractionation and isolation; (ii) investigate molecular interactions using in silico docking studies targeting key enzymes (AChE, esterases, GABA-T); (iii) evaluate histopathological alterations in treated larvae to confirm tissue-level toxicity; and (iv) conduct field-based trials to assess efficacy under natural environmental conditions.

## Conclusion

In conclusion, the hexane extracts of the tested Solanaceae species, particularly tobacco (*N. tabacum*), demonstrated significant larvicidal activity against *Cx. pipiens* and *Ae. aegypti* mosquito larvae, with concentration- and time-dependent toxicity. Biochemical disturbances, including acetylcholinesterase (AChE) inhibition, esterase suppression, and oxidative stress, suggest a possible multi-target mechanism of action, although direct causation requires further investigation. GC–MS analysis highlighted the role of specific classes of phytochemicals, such as alkaloids and fatty acids, in the observed bioactivity. The main contribution of this study lies in its integrated comparative approach, linking phytochemical composition, larvicidal efficacy, and biochemical activity across four Solanaceae genera. However, the study is limited by its in vitro design and the nature of the correlation between phytochemicals and bioactivity. Future research should focus on (1) bioassay-guided fractionation to identify active compounds; (2) field verification under natural conditions; (3) environmental toxicity assessments on non-target organisms; and (4) development of formulations for integrated vector management. Addressing these aspects is essential for the successful transformation of Solanaceae-derived biopesticides into practical vector control tools.

**Table 1:** Efficacy of four hexane plant extracts on *Culex pipiens* and *Aedes aegypti* larval mortality, 24 h post-treatment.

Time (hr)	Solvent	Concentration (ppm)						
		0	50	125	250	500	1000	1500
*Culex pipiens*	*D. stramonium*	0 ± 0^aF^	16 ± 1.63^bE^	39 ± 3.42^bD^	74 ± 2.58^bC^	95 ± 1.00^bB^	100 ± 0^aA^	100 ± 0^aA^
	*C. annuum*	0 ± 0^aF^	12 ± 1.63^cE^	32 ± 1.63^cD^	66 ± 2.00^cC^	90 ± 2.00^cB^	100 ± 0^aA^	100 ± 0^aA^
	*N. tabacum*	0 ± 0^aE^	19 ± 1.91^aD^	43 ± 2.52^aC^	80 ± 2.83^aB^	99 ± 1.00^aA^	100 ± 0^aA^	100 ± 0^aA^
	*W. somnifera*	0 ± 0^aF^	11 ± 1.00^cE^	31 ± 1.91^cD^	63 ± 1.91^dC^	87 ± 3.42^dB^	99 ± 1.00^aA^	100 ± 0^aA^
*Aedes aegypti*	*D. stramonium*	0 ± 0^aG^	13 ± 1.00^aF^	27 ± 1.00^bE^	49 ± 2.52^bD^	72 ± 1.63^bC^	96 ± 3.46^bB^	100 ± 0^aA^
	*C. annuum*	0 ± 0^aG^	10 ± 1.15^bF^	21 ± 1.00^cE^	42 ± 3.46^cD^	65 ± 3.42^cC^	91 ± 1.91^cB^	100 ± 0^aA^
	*N. tabacum*	0 ± 0^aF^	14 ± 1.15^aE^	34 ± 2.58^aD^	57 ± 1.91^aC^	83 ± 1.91^aB^	100 ± 1.15^aA^	100 ± 0^aA^
	*W. somnifera*	0 ± 0^aG^	8 ± 0.00^cF^	18 ± 1.15^dE^	35 ± 1.91^dD^	60 ± 2.31^dC^	86 ± 1.15^dB^	100 ± 0^aA^

a, b & c: There is no significant difference (*P* > 0.05) between any two means, within the same column have the same superscript letter. A, B & C: There is no significant difference (*P* > 0.05) between any two means, within the same row have the same superscript letter.

**Table 2:** Efficacy of four hexane plant extracts on *Culex pipiens* and *Aedes aegypti* larval mortality, 48 h post-treatment.

Time (hr)	Solvent	Concentration (ppm)						
		0	50	125	250	500	1000	1500
*Culex pipiens*	*D. stramonium*	0 ± 0^aE^	21 ± 2.52^bD^	45 ± 1.91^bC^	88 ± 1.63^bB^	100 ± 0.00^aA^	100 ± 0.00^aA^	100 ± 0.00^aA^
	*C. annuum*	0 ± 0^aE^	18 ± 1.15^cD^	40 ± 1.63^cC^	82 ± 2.58^cB^	100 ± 1.15^aA^	100 ± 0.00^aA^	100 ± 0.00^aA^
	*N. tabacum*	0 ± 0^aE^	24 ± 2.31^aD^	54 ± 2.58^aC^	93 ± 2.52^aB^	100 ± 0.00^aA^	100 ± 0.00^aA^	100 ± 0.00^aA^
	*W. somnifera*	0 ± 0^aE^	17 ± 1.91^cD^	41 ± 2.52^cC^	80 ± 1.63^dB^	98 ± 1.15^bA^	100 ± 0.00^aA^	100 ± 0.00^aA^
*Aedes aegypti*	*D. stramonium*	0 ± 0^aF^	15 ± 1.91^aE^	34 ± 2.58^bD^	62 ± 2.00^bC^	91 ± 3.42^bB^	100 ± 0.00^aA^	100 ± 0.00^aA^
	*C. annuum*	0 ± 0^aF^	12 ± 1.63^bE^	31 ± 2.52^cD^	57 ± 1.00^cC^	85 ± 3.00^cB^	100 ± 0.00^aA^	100 ± 0.00^aA^
	*N. tabacum*	0 ± 0^aF^	16 ± 1.63^aE^	38 ± 2.58^aD^	71 ± 4.43^aC^	95 ± 3.00^aB^	100 ± 0.00^aA^	100 ± 0.00^aA^
	*W. somnifera*	0 ± 0^aF^	11 ± 1.00^bE^	28 ± 1.63^dD^	51 ± 1.91^dC^	81 ± 1.91^dB^	100 ± 0.00^aA^	100 ± 0.00^aA^

a, b & c: There is no significant difference (*P* > 0.05) between any two means, within the same column have the same superscript letter. A, B & C: There is no significant difference (*P* > 0.05) between any two means, within the same row have the same superscript letter.

**Table 3:** Lethal concentrations (ppm) of four hexane plant extracts on *Culex pipiens* larval mortality, 24 and 48 h post-treatment.

Time (h)	Treatment	LC_50_ (Low-Up.)	LC_90_ (Low-Up.)	LC_95_ (Low-Up.)	Slope ± SE	X^2^ (sign.)
24	*D. stramonium*	139.36 (121.83–157.95)	428.22 (362.40–527.24)	588.67 (483.03–758.27)	2.628 ± 0.198	4.447 (0.348)
	*C. annuum*	165.31 (145.73–186.19)	492.29 (420.69–596.63)	670.76 (557.49–845.87)	2.704 ± 0.190	5.492 (0.240)
	*N. tabacum*	120.45 (105.71–135.86)	338.60 (289.89–410.74)	453.86 (377.81–574.00)	2.855 ± 0.219	8.164 (0.085)
	*W. somnifera*	176.37 (155.23–198.98)	546.69 (456.79–664.61)	753.38 (623.86–953.72)	2.608 ± 0.180	4.088 (0.394)
48	*D. stramonium*	109.23 (78.02–143.04)	290.17 (229.23–468.65)	382.77 (302.47–674.88)	3.020 ± 0.241	11.129 (0.025)
	*C. annuum*	122.16 (87.53–160.85)	325.37 (256.91–526.55)	429.52 (339.59–756.52)	3.012 ± 0.232	11.680 (0.019)
	*N. tabacum*	95.30 (83.43–107.98)	248.79 (213.76–301.53)	326.56 (272.87–413.20)	3.075 ± 0.258	8.631 (0.071)
	*W. somnifera*	125.85 (110.77–141.69)	348.93 (299.17–422.35)	465.88 (388.60–587.32)	2.893 ± 0.220	6.605 (0.158)

**Table 4:** Lethal concentrations (ppm) of four hexane plant extracts on *Aedes aegypti* larval mortality, 24 and 48 h post-treatment.

Time (h)	Treatment	LC_50_ (Low-Up.)	LC_90_ (Low-Up.)	LC_95_ (Low-Up.)	Slope ± SE	X^2^ (sign.)
24	*D. stramonium*	219.76 (150.32–305.11)	834.43 (630.61–1478.79)	1218.01 (919.20–2382.87)	2.211 ± 0.150	12.868 (0.011)
	*C. annuum*	294.17 (252.21–342.08)	1419.14 (1110.77–1949.04)	2216.96 (1650.37–3270.43)	1.875 ± 0.146	7.679 (0.104)
	*N. tabacum*	176.97 (127.51–234.33)	605.36 (464.98–969.85)	857.88 (651.89–1493.18)	2.399 ± 0.165	10.236 (0.036)
	*W. somnifera*	316.45 (212.04–459.22)	1196.42 (911.74–2338.92)	1744.27 (1241.44–3813.28)	2.218 ± 0.149	15.754 (0.003)
48	*D. stramonium*	160.70 (140.88–181.78)	507.76 (431.69–619.23)	703.55 (580.87–894.75)	2.565 ± 0.180	9.069 (0.059)
	*C. annuum*	182.77 (160.65–206.46)	582.36 (495.03–709.87)	808.84 (667.82–1027)	2.546 ± 0.174	8.996 (0.061)
	*N. tabacum*	141.26 (123.92–159.58)	423.37 (361.17–514.78)	577.90 (579.08–732.50)	2.688 ± 0.185	6.301 (0.177)
	*W. somnifera*	201.87 (142.29–273.83)	647.28 (498.66–1080.36)	900.61 (693.96–1634.55)	2.532 ± 0.171	12.829 (0.012)

**Table 5:** The effect of four hexane plant extracts against the activities of different esterase enzymes in mosquitoes.

Plant extracts	Acetylcholinesterase (AChE) (nmol ACh hydrolyzed/min/mg protein)	α-esterase (α-EST) (mmol/min/mg protein)	β-esterase (β-EST) (mmol/min/mg protein)
Control	13.68 ± 0.06^a^	3.34 ± 0.08^a^	4.48 ± 0.04^a^
*D. stramonium*	7.33 ± 0.05^ab^	1.79 ± 0.07^ab^	2.40 ± 0.03^ab^
*C. annuum*	10.13 ± 0.03^b^	2.47 ± 0.04^b^	3.32 ± 0.02^b^
*N. tabacum*	**5.77 ± 0.05** ^**ab**^	**1.41 ± 0.07** ^**ab**^	**1.89 ± 0.03** ^**ab**^
*W. somnifera*	12.88 ± 0.03^b^	3.00 ± 0.04^b^	3.85 ± 0.02^b^

Values were calculated from three replicates and expressed as mean ± SE; a, b & c: There is no significant difference (*P* > 0.05) between any two means, within the same column have the same superscript letter.

**Table 6:** The effect of four hexane plant extracts against the activities of infection response enzymes in mosquitoes.

Plant extracts	Gamma amino butyric acid transaminase (GABA-T) (units/g tissue)	Amylase (units/g tissue)	Lipase (units/g tissue)
Control	6.47 ± 0.04^a^	18.34 ± 0.03^a^	2.72 ± 0.03^a^
*D. stramonium*	3.47 ± 0.03^ab^	11.51 ± 0.03^ab^	1.68 ± 0.02^ab^
*C. annuum*	5.06 ± 0.02^b^	14.09 ± 0.02^b^	2.04 ± 0.02^b^
*N. tabacum*	**2.73 ± 0.03** ^**ab**^	**7.74 ± 0.03** ^**ab**^	**1.15 ± 0.02** ^**ab**^
*W. somnifera*	5.85 ± 0.02^b7^	16.90 ± 0.02^b^	2.22 ± 0.02^b^

Values were calculated from three replicates and expressed as mean ± SE; a, b & c: There is no significant difference (*P* > 0.05) between any two means, within the same column have the same superscript letter.

**Table 7:** The effect of four hexane plant extracts against markers of the antioxidant defense in mosquitoes.

Plant extracts	SOD (units/g tissue)	G-S-T (units/g tissue)	CAT (U/mg protein/min)	GSH (µg/mg protein)	LPO (nmol/mg protein)	TPC (nmol/mg protein)
Control	3.56 ± 0.04^a^	61.41 ± 0.53^a^	5.32 ± 0.04^a^	2.37 ± 0.02^a^	3.61 ± 0.05^b^	5.63 ± 0.04^b^
*D. stramonium*	1.91 ± 0.03^ab^	32.91 ± 0.43^ab^	2.85 ± 0.03^ab^	1.23 ± 0.01^ab^	6.74 ± 0.06^ab^	10.50 ± 0.05^ab^
*C. annuum*	2.17 ± 0.02^b^	37.57 ± 0.27^b^	3.26 ± 0.02^b^	1.45 ± 0.01^b^	5.90 ± 0.10^a^	9.20 ± 0.08^a^
*N. tabacum*	**1.50 ± 0.03** ^**ab**^	**25.91 ± 0.43** ^**ab**^	**2.25 ± 0.03** ^**ab**^	**0.97 ± 0.01** ^**ab**^	**8.56 ± 0.06** ^**ab**^	**13.34 ± 0.05** ^**ab**^
*W. somnifera*	2.63 ± 0.02^b^	45.45 ± 0.27^b^	3.94 ± 0.02^b^	1.76 ± 0.01^b^	4.88 ± 0.10^a^	7.60 ± 0.08^a^

Values were calculated from three replicates and expressed as mean ± SE; a, b & c: There is no significant difference (*P* > 0.05) between any two means, within the same column have the same superscript letter.

**Table 8:** Volatile metabolites identified from the *n*-hexane extracts of the four Solanaceous plants (Capsicum, Tobacco, Datura and *Withani*a).

No.	Component	*R*_t_ (min.)	RI*		% Composition				Method of Identification	Molecular Formula
			Cal.	Rep.	Cap.	To.	Dat.	Wt.		
1	1,2,4-trimethyl-Cyclopentane	3.083	**763**	**777**	-	-	-	1.40	RI/MS	C_8_H_16_
2	(1*α*,2*α*,3*β*)−1,2,3-trimethyl- Cyclopentane	3.181	**767**	**-**	-	-	-	1.08	RI/MS	C_8_H_16_
3	4-methyl-Heptane	3.386	**775**	**770**	-	-	-	0.91	RI/MS	C_8_H_18_
4	2-methyl-Heptane	3.478	**778**	**778**	-	-	-	**5.65**	RI/MS	C_8_H_18_
5	3-methyl-Heptane	3.605	**783**	**783**	-	-	-	**2.44**	RI/MS	C_8_H_16_
6	*cis*−1,3-dimethyl-Cyclohexane	3.646	**785**	**778**	-	-	-	**6.55**	RI/MS	C_8_H_16_
7	1,4-dimethyl-Cyclohexane	3.675	**786**	**782**	-	-	-	**2.68**	RI/MS	C_8_H_16_
8	3-Hexanol	3.740	**788**	**784**	-	-	-	0.49	RI/MS	C_6_H_14_O
9	1,1-dimethyl-Cyclohexane	3.770	**789**	**783**	-	-	-	0.51	RI/MS	C_8_H_16_
10	4-methyl-2-Pentanol	3.822	**791**	**790**	-	-	-	0.80	RI/MS	C_6_H_14_O
11	*cis*−1-ethyl-2-methyl-Cyclopentane	3.891	**794**	**790**	-	-	-	0.61	RI/MS	C_8_H_16_
12	*trans*−1,3-dimethyl-Cyclohexane	3.993	**798**	**798**	-	-	-	1.30	RI/MS	C_8_H_16_
13	Octane	4.107	**802**	**800**	-	-	-	**16.91**	RI/MS	C_8_H_16_
14	hexyl-Cyclopentane	4.270	**809**	**-**	-	-	-	0.04	RI/MS	C_11_H_22_
15	*cis*−1,2-dimethyl-Cyclohexane	4.611	**822**	**826**	-	-	-	0.54	RI/MS	C_8_H_16_
16	ethyl-Cyclohexane	4.729	**826**	**827**	-	-	-	1.60	RI/MS	C_8_H_16_
17	Cyclogeraniolane	4.855	**831**	**839**	-	-	-	0.33	RI/MS	C_9_H_18_
18	2,5-dimethyl-Heptane	4.907	**833**	**833**	-	-	-	0.29	RI/MS	C_9_H_20_
19	Ethylbenzene	5.155	**843**	**843**	-	-	-	0.54	RI/MS	C_8_H_10_
20	1,3,5-trimethyl-Cyclohexane	5.216	**845**	**-**	-	-	-	0.20	RI/MS	C_9_H_18_
21	octahydro-Pentalene	5.482	**855**	**861**	-	-	-	0.14	RI/MS	C_8_H_14_
22	3-methyl-Octane	5.817	**868**	**852**	-	-	-	0.28	RI/MS	C_9_H_20_
23	1-Ethyl-4-methylcyclohexane	6.129	**881**	**883**	-	-	-	0.19	RI/MS	C_9_H_18_
24	Cyclohexane propanol	6.346	**919**	**-**	1.22	-	-	-	RI/MS	C_9_H_18_O
25	2,2,3,3-tetramethyl-Pentane	6.515	**925**	**-**	1.00	-	-	-	RI/MS	C_9_H_20_
26	Nonane	6.596	**898**	**900**	-	-	-	0.59	RI/MS	C_13_H_28_
27	propyl-Cyclohexane	6.647	**929**	**926**	**2.47**	-	-	-	RI/MS	C_9_H_18_
28	(*E*)−6-methyl-3-Undecene	6.981	**940**	**-**	1.11	-	-	-	RI/MS	C_12_H_24_
29	(R)-(-)-(*Z*)−14-Methyl-8-hexadecen-1-ol	7.119	**944**	**-**	0.29	-	-	-	RI/MS	C_17_H_34_O
30	*trans*−2-ethyl-1,1’-*bi*-cyclohexyl	7.245	**948**	**-**	0.18	-	-	-	RI/MS	C_14_H_26_
31	1,1,2,3-tetramethyl-Cyclohexane	7.391	**953**	**-**	**2.66**	-	-	-	RI/MS	C_10_H_20_
32	1R-*α*-Pinene	7.415	**925**	**929**	-	-	-	0.03	RI/MS	C_10_H_16_
33	Mesitylene	7.848	**967**	**962**	1.87	-	-	-	RI/MS	C_9_H_12_
34	3-methyl-Nonane	7.899	**969**	**962**	**4.63**	-	-	-	RI/MS	C_10_H_22_
35	4-phenyl-Valeric acid	8.190	**978**	**-**	0.43	-	-	-	RI/MS	C_11_H_14_O_2_
36	*trans*−1-methyl-4-(1-methylethyl)-Cyclohexane	8.245	**980**	**976**	1.12	-	-	-	RI/MS	C_10_H_20_
37	1-methyl-3-propyl-Cyclohexane	8.325	**983**	**-**	1.24	-	-	-	RI/MS	C_10_H_20_
38	Hemimellitene	8.617	**992**	**999**	**3.33**	-	-	-	RI/MS	C_9_H_12_
39	*di*-Hydrocapsaicin	8.806	**998**	**-**	**14.31**	-	-	-	RI/MS	C_18_H_29_NO_3_
40	*β*-Myrcene	9.111	**979**	**975**	-	-	-	0.10	RI/MS	C_10_H_16_
41	Hydratropic acid, oct-3-en-2-yl ester	9.140	**1009**	**-**	0.24	-	-	-	RI/MS	C_17_H_24_O_2_
42	5-ethyl-2-methyl-Heptane	9.512	**1021**	**-**	**3.26**	-	-	-	RI/MS	C_10_H_22_
43	butyl-Cyclohexane	9.771	**1029**	**1026**	1.03	-	-	-	RI/MS	C_10_H_20_
44	3,7-dimethyl-Nonane	10.016	**1037**	**1042**	0.45	-	-	-	RI/MS	C_11_H_24_
45	*β*-Phellandrene	10.178	**1013**	**1013**	-	-	-	0.33	RI/MS	C_10_H_16_
46	D-Limonene	10.241	**1015**	**1018**	-	-	-	0.77	RI/MS	C_10_H_16_
47	(*Z*)-Dec-4-en-1-yl propyl carbonate	10.470	**1051**	**-**	1.70	-	-	-	RI/MS	C_14_H_26_O_3_
48	2,5-dimethyl-Nonane	10.611	**1056**	**1059**	1.52	-	-	-	RI/MS	C_11_H_24_
49	1-ethyl-3,5-dimethyl-Benzene	10.660	**1058**	**1058**	0.59	-	-	-	RI/MS	C_10_H_14_
50	4-methyl-Decane	10.712	**1059**	**1051**	0.93	-	-	-	RI/MS	C_11_H_24_
51	2-methyl-Decane	10.821	**1063**	**1053**	1.87	-	-	-	RI/MS	C_11_H_24_
52	3-methyl-Decane	11.025	**1069**	**1069**	0.85	-	-	-	RI/MS	C_11_H_24_
53	4-ethyl-1,2-dimethyl-Benzene	11.250	**1076**	**1075**	0.60	-	-	-	RI/MS	C_10_H_14_
54	1-isopropyl-1-methyl-Cyclohexane	11.423	**1082**	**-**	0.80	-	-	-	RI/MS	C_10_H_20_
55	*p*-Cymene	11.521	**1085**	**-**	0.91	-	-	0.09	RI/MS	C_10_H_14_
56	4,6-dimethyl-Dodecane	11.568	**1057**	**-**	-	-	-	0.11	RI/MS	C_14_H_30_
57	Capsaicin	11.950	**1099**	**-**	**15.46**	-	-	-	RI/MS	C_18_H_27_NO_3_
58	1,3-diethyl-5-methyl-Benzene	12.091	**1103**	**-**	0.66	-	-	-	RI/MS	C_11_H_16_
59	*trans*−2-methyl-Decalin	12.217	**1124**	**1138**	0.94	-	-	-	RI/MS	C_11_H_20_
60	(1α,2*β*,4α.)−1,4-dimethyl-2-(2-methylpropyl)-, Cyclohexane	12.342	**1081**	**-**	-	-	-	0.50	RI/MS	C_12_H_24_
61	*trans*-Verbenyl isovalerate	12.468	**1116**	**-**	1.02	-	-	-	RI/MS	C_15_H_24_O_2_
62	1,2,3,4-tetramethyl-Benzene	12.590	**1119**	**-**	0.38	-	-	-	RI/MS	C_10_H_14_
63	3,7-dimethyl-Decane	12.794	**1126**	**1127**	0.44	-	-	-	RI/MS	C_12_H_26_
64	*trans*−1-ethyl-1,3-dimethyl-Cyclohexane	12.802	**1095**	**-**	-	-	-	0.18	RI/MS	C_10_H_20_
65	1,3-dimethyl-Adamantane	12.938	**1099**	**-**	-	-	-	**15.04**	RI/MS	C_12_H_20_
66	pentyl-Cyclohexane	13.002	**1133**	**1134**	0.86	-	-	-	RI/MS	C_11_H_22_
67	1-methyl-3-propyl-Cyclooctane	13.255	**1109**	**-**	-	-	-	0.09	RI/MS	C_12_H_24_
68	1,3,5-trimethyl-Adamantane	13.355	**1113**	**-**	-	-	-	0.38	RI/MS	C_13_H_22_
69	1,1’-*bi*-Cyclooctyl	13.465	**1116**	**-**	-	-	-	0.09	RI/MS	C_16_H_30_
70	2,4-diethyl-1-methyl-Cyclohexane	13.521	**1118**	**-**	-	-	-	0.14	RI/MS	C_11_H_22_
71	1,2,3,5-tetramethyl-Benzene	13.600	**1152**	**1151**	0.30	-	-	-	RI/MS	C_10_H_14_
72	Sulfurous acid, decyl 2-ethylhexyl ester	13.678	**1154**	**-**	0.83	-	-	-	RI/MS	C_18_H_38_O_3_S
73	10-Methyl-*Z*−11-tridecen-1-ol acetate	13.720	**1124**	**-**	-	-	-	0.20	RI/MS	C_16_H_30_O_2_
74	5-(1-methylpropyl)-Nonane	13.801	**1158**	**-**	0.36	-	-	-	RI/MS	C_13_H_28_
75	Hexadecane	13.933	**1163**	**-**	1.73	-	-	-	RI/MS	C_16_H_34_
76	1,1-*bis*-(dodecyloxy)-Hexadecane	13.956	**1132**		-	-	-	0.30	RI/MS	-
77	(1*α*,2*α*,4*α*,5*α*)−1,2,4,5-tetraethyl-Cyclohexane	14.090	**1136**	**-**	-	-	-	0.08	RI/MS	C_14_H_28_
78	3-methyl-Undecane	14.138	**1169**	**1169**	0.68	-	-	-	RI/MS	C_12_H_26_
79	1-methyl-4-(1-methylbutyl)-Cyclohexane	14.233	**1141**	**-**	-	-	-	0.21	RI/MS	C_12_H_24_
80	*cis*,*cis*,*cis*−1-Isobutyl-2,5-dimethylcyclohexane	14.361	**1145**	**1137**	-	-	-	0.19	RI/MS	C_12_H_24_
81	*decahydro*−2,6-dimethyl-Naphthalene	14.648	**1154**	**-**	-	-	-	**5.01**	RI/MS	C_12_H_22_
82	*decahydro*−2,3-dimethyl-Naphthalene	15.000	**1165**	**-**	-	-	-	0.72	RI/MS	C_12_H_22_
83	1-ethyl-Cyclohexene	15.038	**1167**	**-**	-	-	-	0.44	RI/MS	C_8_H_14_
84	Dodecane	15.039	**1198**	**-**	**8.27**	-	-	-	RI/MS	C_14_H_30_
85	6-methyl-Tridecane	15.454	**1212**	**-**	1.04	-	-	-	RI/MS	C_14_H_30_
86	*decahydro*−1,5-dimethyl-Naphthalene	15.546	**1183**	**-**	-	-	-	1.10	RI/MS	C_12_H_22_
87	1-(2-methyl-2-cyclopenten-1-yl)-Cyclohexene	15.883	**1194**	**-**	-	-	-	1.04	RI/MS	C_12_H_18_
88	Tritetracontane	16.931	**1264**	**-**	**4.18**	-	-	0.20	RI/MS	-
89	Tridecane	17.986	**1301**	**1300**	0.78	-	-	-	RI/MS	C_14_H_30_
90	Acetic acid, [4-(1-hydroxy-1-methylethyl)cyclohex-1-enyl]methyl ester	18.226	**1309**	**-**	-	-	1.49	-	RI/MS	C_12_H_20_O_3_
91	(S)−3-(1-methyl-2-pyrrolidinyl)-Pyridine	19.411	**1351**	**1341**	-	**85.96**	**4.00**	-	RI/MS	C_10_H_14_N_2_
92	(1 S,3R,5 S,6R)-(-)−5-Caranol	21.628	**1432**	**-**	-	-	1.04	-	RI/MS	C_10_H_18_O
93	Sulfurous acid, hexyl octyl ester	23.259	**1496**	**-**	0.29	-	-	-	RI/MS	C_14_H_30_O_3_S
94	*trans*-*p*-Menth-8-ene-1,2-diol	23.485	**1505**	**-**	-	-	**2.22**	-	RI/MS	C_10_H_18_O_2_
95	2,4-*bis*-(1,1-dimethylethyl)-Phenol	23.810	**1518**	**-**	0.35	-	-	-	RI/MS	C_14_H_22_O
96	Heneicosane	24.284	**1495**	**-**	-	-	-	1.13	RI/MS	C_21_H_44_
97	Tetradecanoic acid	29.699	**1766**	**1769**	-	-	1.95	-	RI/MS	C_14_H_28_O_2_
98	Hexadecanoic acid, methyl ester	33.120	**1927**	**1927**	-	1.21	**4.11**	-	RI/MS	C_17_H_34_O_2_
99	Eicosanoic acid	33.888	**1965**	**-**	**2.21**	-	-	-	RI/MS	C_20_H_40_O_2_
100	*n*-Hexadecanoic acid	33.924	**1966**	**1968**	-	**6.77**	**53.81**	-	RI/MS	C_16_H_32_O_2_
101	9,12-Octadecadienoic acid, methyl ester	36.463	**2097**	**2094**	-	0.37	-	-	RI/MS	C_19_H_34_O_2_
102	(*E*)−9-Octadecenoic acid, methyl ester	36.568	**2103**	**2085**	-	-	**3.26**	-	RI/MS	C_19_H_36_O_2_
103	Cyclopropaneoctanoic acid, 2-[[2-[(2-ethylcyclopropyl)methyl]cyclopropyl]methyl]-, methyl ester	36.585	**2104**	**-**	-	0.62	-	-	RI/MS	C_22_H_38_O_2_
104	Methyl stearate	37.051	**2128**	**2128**	-	0.19	1.50	-	RI/MS	C_19_H_38_O_2_
105	Oleic acid	37.317	**2142**	**2141**	0.63	1.91	**20.29**	-	RI/MS	C_18_H_34_O_2_
106	9-Hexadecenoic acid	37.428	**2148**	**-**	-	1.09	-	-	RI/MS	C_16_H_30_O_2_
107	Octadecanoic acid (stearic acid)	37.751	**2165**	**2167**	-	0.82	**4.25**	-	RI/MS	C_18_H_36_O_2_
108	4-methylene-1-methyl-2-(2-methyl-1-propen-1-yl)−1-vinyl-Cycloheptane	37.965	**2177**	**-**	-	0.26	-	-	RI/MS	C_15_H_24_
109	Scopolamine	41.502	**2378**	**-**	-	-	0.44	-	RI/MS	C_17_H_21_NO_4_
110	Vitamin E	52.796	**3149**	**3149**	0.54	-	-	-	RI/MS	C_29_H_50_O_2_
	**% Identification** **No. of compounds**				**92.56** **(48)**	**99.20** **(10)**	**98.36** **(12)**	**74.54** **(49)**		

*R*_**t**_: retention time, *RI*: retention index, *Dat.*: *Datura stramonium*, *Cap.*: *Capsicum annuum*, **To.**: *Nicotiana tabacum*,* Wt.*: *Withania somnifera.* The percentage values reported in each plant column represent independent GC–MS analyses of the corresponding extract. A dash (–) indicates that the compound was not detected in that particular extract. * (RI): Retention Index.

## Supplementary Information

Below is the link to the electronic supplementary material.

Supplementary Material 1

## Data Availability

The datasets used and/or analyzed during the current study available from the corresponding author on reasonable request. Correspondence: mohamed.albaz@fsc.bu.edu.eg.
