# Supplementary material for: Comparative larvicidal efficacy and phytochemical profiling of selected Solanaceous hexane extracts against Culex pipiens and Aedes aegypti
Source: Sci Rep. 2026 Sep 5;16:27824. doi: 10.1038/s41598-026-68059-8 (PMC13546292; doi:10.1038/s41598-026-68059-8)
Supplement: Supplementary file 1 [file 41598_2026_68059_MOESM1_ESM.docx]

**Suppl. Fig.1. GC/MS chromatogram for Capsicum *n*-hexane extract**

**Suppl. Fig.2. GC/MS chromatogram for Datura *n*-hexane extract**

**Suppl. Fig.3. GC/MS chromatogram for Tobacco *n*-hexane extract**

**Suppl. Fig.4. GC/MS chromatogram for Withania *n*-hexane extract**
